# Occurrence and Characteristics of Microplastics in Wild and Farmed Shrimps Collected from Cau Hai Lagoon, Central Vietnam

**DOI:** 10.3390/molecules28124634

**Published:** 2023-06-08

**Authors:** Tran Thi Ai My, Nguyen Duy Dat, Nguyen Quoc Hung

**Affiliations:** 1 Department of Chemistry, University of Sciences, Hue University, Hue 53000, Vietnam; 2 Faculty of Chemical & Food Technology, Ho Chi Minh City University of Technology and Education, Thu Duc, Ho Chi Minh 70000, Vietnam; datnd@hcmute.edu.vn; 3 Center of Analytical Sevices and Experimentation HCMc, Ho Chi Minh 70000, Vietnam; ngqhung2005@gmail.com

**Keywords:** gastrointestinal tract, tissue, microplastics, wild-caught shrimps, farmed shrimps

## Abstract

This study investigated the occurrence of microplastics (MPs) in the gastrointestinal tracts (GT) and tissues of four common shrimps (including two wild-caught shrimps and two farmed shrimps) collected from a high-diversity lagoon in central Vietnam. The numbers of MP items in greasy-back shrimp (*Metapenaeus ensis*), green tiger shrimp (*Penaeus semisulcatus*), white-leg shrimp (*Litopenaeus vannamei*), and giant tiger shrimp (*Penaeus monodon*), determined per weight and individual, were 0.7 ± 0.3, 0.6 ± 0.2, 1.1 ± 0.4, and 0.5 ± 0.3 (items/g-ww), and 2.5 ± 0.5, 2.3 ± 0.7, 8.6 ± 3.5, 7.7 ± 3.5 (items/individual), respectively. The concentration of microplastics in the GT samples was significantly higher than that in the tissue samples (*p* < 0.05). The number of microplastics in the farmed shrimp (white-leg shrimp and black tiger shrimp) was statistically significantly higher than the number of microplastics in the wild-caught shrimp (greasy-back and green tiger shrimps) (*p* <0.05). Fibers and fragments were the dominant shapes of the MPs, followed by pellets, and these accounted for 42–69%, 22–57%, and 0–27% of the total microplastics, respectively. The chemical compositions determined using FTIR confirmed six polymers, in which rayon was the most abundant polymer, accounting for 61.9% of the MPs found, followed by polyamide (10.5%), PET (6.7%), polyethylene (5.7%), polyacrylic (5.8%), and polystyrene (3.8%). As the first investigation on the MPs in shrimps from Cau Hai Lagoon, central Vietnam, this study provides useful information on the occurrences and characteristics of the microplastics in the gastrointestinal tracts and tissues of four shrimp species that live in different living conditions.

## 1. Introduction

More than five trillion plastic pieces (about 250,000 tons) have been found in the world’s oceans [1]. According to Plastic Europe’s annual assessment report for 2017–2018 [2], the rising production and consumption of plastic have led to an increase of 8.4 million tons of plastic trash being discharged into the marine environment. Large quantities of plastic debris from the continent reach the marine environment primarily through rivers [3], industrial and urban wastewater, and runoff from beach sediments and their adjacent fields [4]. Other direct sources of this plastic debris in the marine environment include offshore industrial activities (such as oil and gas extraction), aquaculture activities, lost fish nets, and littering at sea, including tourism-related activities [4]. Microplastics (MPs) are small (items smaller than 5.0 mm), non-biodegradable, and persistent polymers, which are pervasive in the environment and have raised many concerns about their effects on human health, biodiversity, and ecosystem function worldwide [5,6,7,8,9,10,11]. Moreover, some studies have indicated that MPs in the environment can act as media for adsorbing heavy metals or micro-pollutants, which are more toxic [12,13].

Vietnam has a high potential for MP contamination due to its rapid economic development and inadequate waste management. However, limited information regarding the occurrences of MPs in environmental matrices has been reported in Vietnam. Therefore, studies on the occurrences of MPs in environmental compartments and marine creatures are necessary, which will help to raise public consciousness about MP pollution and encourage people to take action to reduce their MP discharges. A previous study estimated that Vietnam has the world’s fourth largest amount of plastic waste, which originates from the land and is deposited into the ocean, with 1.83 million metric tons being deposited per year [14]. High levels of plastics and MPs were found in the Saigon River, which flows through Ho Chi Minh City—one of the most developed cities in Vietnam [14]. According to daily analyses of the garbage pulled from the Saigon River, between 11 and 43 percent of the trash in the river is made of plastic. Furthermore, it was estimated that, daily, each person discharges from 0.96 to 19. 9 g of plastic debris into the river, which means that, yearly, every person releases from 350 to 7270 g of plastic into the river [14].

The decapod shrimp is found in shallow coastal and estuary environments with soft bottoms such as sand and mud, where research has indicated a high buildup of microplastics [15,16,17,18]. This means that the shrimp may be subjected to varying quantities of microplastics at various points throughout their lifecycle. Greasy-back shrimp, green tiger shrimp, white-leg shrimp, and giant tiger shrimp are common species that are wild-caught and farmed in Cau Hai Lagoon, consumed mainly by people living not only in Thua Thien Hue, but also the central Vietnam region. Therefore, a high risk to human health is posed when people eat shrimp containing MPs, with or without adsorbed contaminants on the items [19,20]. Surprisingly, aquaculture products can also be contaminated by MPs due to the consumption of food containing MPs [21]. Therefore, MPs and the toxins that they absorb can move up the food chain and accumulate at higher trophic levels, including in humans [19,21].

Cau Hai Lagoon is part of the Tam Giang-Cau Hai ecosystem—the largest coastal lagoonal system of southeast Asia, with an area of 216 km^2^ and a high biodiversity [22]. This lagoon ecosystem feeds 500,000 people in 44 communes in five regions and towns around the lagoon. The rapid expansion of coastal residential areas, aquaculture regions, industrial zones, and small enterprises in the basin, as well as tourists, pose a high pollution burden on its surrounding ecosystems, including the Cau Hai Lagoon, especially with plastic waste. Aquaculture and fishing, especially shrimp, are the important economic sectors of the people living around Cau Hai Lagoon; therefore, an analysis of the microplastics in shrimp collected from this area is necessary, in order to raise concerns about the potential impacts of these microplastics on the health of both the seafood industry and its consumers. This study aimed to characterize the occurrence of microplastics accumulated in the four most consumed shrimps in Cau Hai Lagoon, central Vietnam. These shrimp species included two wild shrimps (greasy-back shrimp and green tiger shrimp) and two farmed shrimps (white-leg shrimp and giant tiger shrimp). The gastrointestinal tracts (GT) and tissues of these wild and farmed shrimps were also separated to analyze the microplastics and explore the differences in the abundance and morphological properties of these MPs. Investigating the microplastics in shrimp can provide valuable information, not only for further understanding the accumulation of MPs in shrimps, but also for adding the level of these MPs accumulated in aquatic creatures in Vietnam to those reported worldwide. 

## 2. Results

### 2.1. Abundance of Microplastics 

The number of MPs found in the samples is summarized in Table 1. MPs were found in 30% of the tissue samples of the wild-caught shrimps (*Metapenaeus ensis* and *Penaeus semisulcatus*), while the detection frequencies of the MPs in the tissue samples of *Penaeus monodon Fabricius*, and *Litopenaeus vannamei* were 83% and 93%, respectively. These results also indicated that 100% of the GT samples of all the shrimps had accumulated MPs. The mean numbers of the MPs extracted from *Metapenaeus ensis* (greasy-back shrimp) and *Penaeus semisulcatus* (green tiger shrimp) were 2.5 ± 0.5 (0–4) items/individual (0.7 ± 0.3 2 items/g-ww) and 2.3 ± 0.7 (0–4) items/individual (0.6 ± 0.2 items/g-ww), respectively. The mean numbers of the microplastics in the whole bodies of the farmed shrimps, including *Litopenaeus vannamei* (white-leg shrimp) and *Penaeus monodon* (giant tiger shrimp), were 8.6 ± 3.5 (1.1 ± 0.4 item/g-ww) and 7.7 ± 3.5 items/individual (05 ± 0.3 item/g-ww), respectively. These figures were calculated from the MP numbers found in the tissue and GT samples of each species. The numbers of MPs accumulated in the tissues of *Metapenaeus ensis*, *Penaeus semisulcatus*, *Litopenaeus vannamei*, and *Crangon crangon* were 0.5 ± 0.4, 0.3 ± 0.3, 0.3 ± 0.1, and 0.2 ± 0.1 (items/g-ww), respectively, while the numbers of items found in the GTs of *Metapenaeus ensis, Penaeus semisulcatus, Litopenaeus vannamei*, and *Crangon crangon* were 2.5 ± 0.5, 3.2 ± 0.7, 104 ± 73, and 28.3 ± 5.7 (items/g-ww), respectively. 

### 2.2. Morphological Characteristics of Microplastics in Shrimps

**Figure 1:**
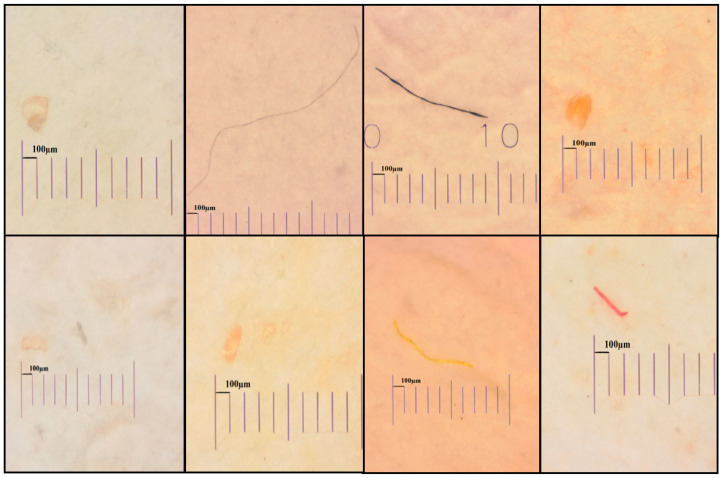
Different shapes of microplastics in shrimps collected from Cau Hai Lagoon, central Vietnam. The photographs were taken directly on the filter paper.

**Figure 2:**
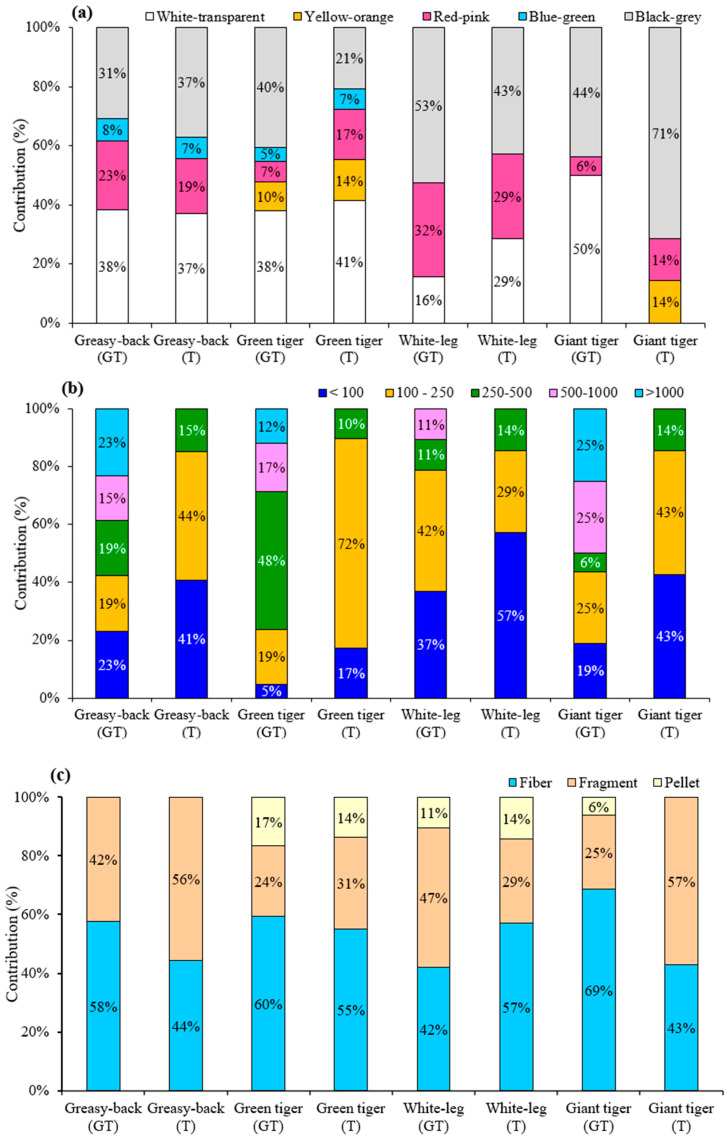
The composition of MP colour (**a**), size (**b**), and shape (**c**) found in the shrimps from Cau Hai Lagoon, central Vietnam.

Figure 1 depicts several forms of MP, including the fibers, fragments, and pellets isolated from the different samples in this study (including tissues and gastrointestinal tracts). As also indicated in Figure 2, the common colors of these fibers and fragments were white, black, red, blue, and transparent, which were widely found in the aquatic creatures [28,29]. The color and size distribution of the MPs found in these four study shrimps are depicted in Figure 2a, which indicates that white-transparent and black-grey were the main MP proportions in the tissues and GTs of these shrimps, accounting for more than 60% of the total MPs, followed by red-pink and blue-green. Yellow-orange was also found in the tissues of giant tiger shrimp and GTs and tissues of green tiger shrimp. 

The size distribution of the MPs extracted from the tissue and GT samples is depicted in Figure 2b, which reveals that the MP sizes found in the tissue samples were smaller than 500 μm, while larger sizes of MPs were found in the GT samples. Figure 2b also indicates that 86–90% of the total MPs in the tissue samples were smaller than 250 μm. On the other hand, MPs larger than 250 μm were dominant in the GT samples, accounting for 57%, 77%, and 56% in the greasy-back, green tiger, and giant tiger shrimps, respectively.

Figure 2c depicts the shape distribution of the MPs in the four kinds of shrimps and indicates that fiber was the most common shape of these MPs observed in both the tissue and GT samples, accounting for 42% to 69% of the total MPs. Fragment was the second most common shape, which ranged from 25% to 57% of the total MPs. Moreover, pellet was not found in all the samples of greasy-back shrimp and tissues of giant tiger shrimp. Pellet was found in the GTs of giant tiger shrimp (6%), while it accounted for 14% and 11% in the tissues and GTs of white-leg shrimp, respectively, and about 17% and 14% in the tissues and GTs of green tiger shrimp, respectively.

### 2.3. Identification of Microplastics in Shrimps with ATR-FTIR

**Figure 3:**
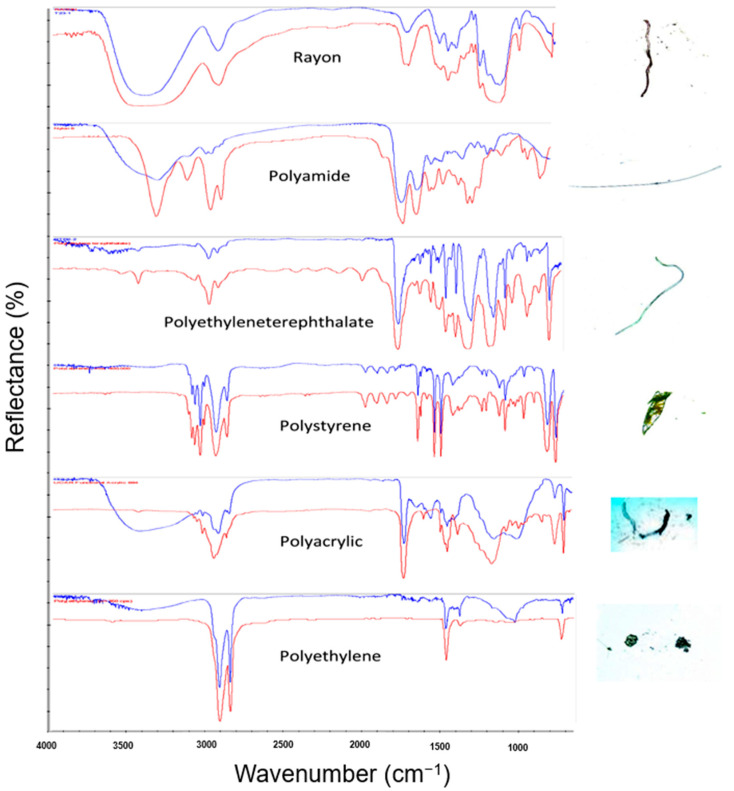
The FTIR spectra of representative microplastic polymers including rayon, polyethylene terephthalate (PET), polyamide, polyethylene, polyacrylic, and polystyrene.

As mentioned in the previous section, a relatively high level of MPs was found in the GTs and tissues of the shrimps collected in Cau Hai Lagoon, central Vietnam. It is essential to identify their chemical compositions, because this could provide valuable information on the sources of the microplastics digested by these shrimps. A total of 268 samples were found in four shrimp species; then, 105 items (39%) were randomly selected for structural determination using ATR-FTIR. Among them, 98 items (93.3%) were confirmed as plastics, comprising 6 polymer types, as summarized in Table 2 and Figure 3. Rayon was the most abundant polymer, accounting for 61.9% of the total MPs, followed by polyamide (10.5%), PET (6.7%), and polyethylene (5.7%). Polystyrene and polyacrylic were also found in the samples in quantities of 3.8% and 5.8%, respectively. 

## 3. Discussion

### 3.1. Abundance of MPs in Shrimp Samples

To the best of our knowledge, this study is the first report on the characteristics of the MPs in the tissues and gastrointestinal tracts of four lagoon shrimp species, including greasy-back shrimp (*Metapenaeus ensis*), green tiger shrimp (*Penaeus semisulcatus*), white-leg shrimp (*Litopenaeus vannamei*), and giant tiger shrimp (*Penaeus monodon*), which were collected from Thua Thien Hue, central Vietnam. The results show that all the wild and farmed shrimps had accumulated MPs in their gastrointestinal tracts and tissues. The detection rate of the MPs in the samples of this study was relatively higher than that reported worldwide [26,29]. Wang et al. [30] investigated the occurrence of MPs in river shrimps in China (*Exopalaemon modestus* and *Macrobrachium nipponense*) and indicated that the detection rates were 50.1% and 64.6%, respectively. Nan et al. [26] also reported that 36.2% of *Paratya australiensis* samples collected in Australia had accumulated MPs. This result corroborates well with the quite high detection rate of MPs in the shrimp collected from central Vietnam, implying a high microplastic accumulation rate in the seafood in central Vietnam. 

Table 2 compares the results obtained in this study with those reported by previous studies. Severini et al. [31] also reported that 1.31 fibers/g were found in commercial shrimp *Pleoticus muelleri* collected in the southwestern Atlantic Ocean, while Devriese et al. [15] found a relatively low level of MPs in European brown shrimp collected in the Southern North Sea, with 1.23 ± 0.99 items/individual (0.68 ± 0.55 items/g). A higher abundance of MPs has been found in other studies, such as 3.40 ± 1.23 and 3.87 ± 1.05 items/g for giant tiger shrimp and brown shrimp in the northern Bay of Bengal, Bangladesh [16]; Nan et al. [26] investigated the microplastics in Australian glass shrimp (*Paratya australiensis*), which commonly live in freshwater bodies. Their results showed that 36% of these shrimps had microplastics at 0.52 ± 0.55 items/individual (24 ± 31 items/g). Gurjar et al. [18] reported that an extremely high level of MPs was found in three species of shrimp collected from the Arabian Sea. Curren et al. [17] reported MPs in three kinds of shrimp (*Litopenaeus vannamei*, *Pleoticus muelleri*, and *Fenneropenaeus indicus*) collected in Singapore, with the highest number of these microplastics reaching 21 ± 4 items per individual. These figures indicate that the levels of MPs found in this study are relatively lower than those reported for the MPs in shrimp collected in other countries.

The number of microplastics in the farmed shrimp (white-leg shrimp and black tiger shrimp) was statistically significantly higher than the number of microplastics in the wild-caught shrimp (greasy-back shrimp and green tiger shrimp) (*p* < 0.05). This finding is in line with that reported by Yan et al. [32], who found a higher abundance of MPs in farmed shrimp compared to mostly wild aquatic creatures. Although all the wild and farmed shrimps lived in Cau Hai Lagoon, they might have been exposed to different water qualities and stagnant water conditions in farming areas and the wild environment, which are associated with aquatic contaminants, including microplastics [33]. It is noted that the detection frequency of MPs in the farmed shrimps was also higher than that in the wild shrimps. This finding also raises the possibility that the increasing accumulation of microplastics in the farmed shrimp may have its origins in the contaminated feed used for growing these farmed shrimps. A similar result was also reported by Murphy [34], with a significantly higher number of MPs being found in farmed seafood (blue mussels and pacific oysters) compared to wild seafood. Except for greasy-back shrimp (*Metapenaeus ensis*), the number of MPs in the GTs of the three other species was significantly higher than that in the tissues (*p* < 0.05), for both MPs/individual and MPs/g-w. A higher occurrence of MPs found in the gastrointestinal system compared to the tissues and other organs has been reported for different aquatic creatures [35]. Valencia-Castañeda et al. [27] investigated the MPs in *Litopenaeus vannamei* and indicated that number of MPs in the different organs of the shrimp followed the order of: gastrointestinal tract > gills > exoskeleton. Atamanalp et al. [36] also investigated the MPs in different tissues of *Mullus barbatus* and *Alosa immaculata* and reported that the MPs accumulated in the different samples followed the order of: gastrointestinal tract > gill > tissue > brain. These results suggest that the main route of shrimp exposure to MPs might be food digestion and feeding them contaminated food could be the main reason for the higher MP levels in farmed shrimps.

### 3.2. Morphological Characteristics of MPs

In terms of the shape of the MPs, the fibrous shape was dominant, which is in line with findings for different aquatic creatures [15,16,17,18,24,25,26]. The portion of the fibrous shape found in this study is comparable with that reported worldwide, such as 70% in fishes from the Mediterranean Sea [37], 68% in fishes from the English Channel [38], 66% in fishes off the Portuguese coast [39], 71% in fishes off the Spanish coast [40], and 83% in fishes off the Spanish coast [41]. The possible sources of these fibers in the coastal and marine waters of Bangladesh could originate from fishing nets, ropes, lines, laundry, and urban wastes, which are similar to other findings. In general, the main shape of the MPs was fibrous, suggesting that the source of these MPs was probably from man-made fibers or fishing nets, or even wastewater from domestic activities such as cloth washing. In addition, fibrous MPs might originate from the plastic rope used in fishing nets or plastic lines where farmed shrimps are grown [42]. 

The results also indicated that all the MPs found in the tissue samples were smaller than 500 µm and 86% to 90% of the MPs were smaller than 250 µm, whereas the sizes of the MPs found in the GTs were more diverse, with these sizes ranging from <100 µm to >1000 µm. Except for the GTs of the white-leg shrimp, the GTs of the other shrimps were accumulated with 54–77% of MPs larger than 500 µm. Yan et al. [32] reported that MPs smaller than 500 μm were dominant (60.6%) in *Litopenaeus vannamei*, while 70% of the total MPs found in *Fenneropenaeus indicus* (Penaeus indicus) were in size range of 500–1000 μm [25] Hossain et al. [16] indicated that the size distribution of MPs in shrimps varied with the species and sampling sites of the shrimp. They also found that the size of the MPs found in the GTs of tiger shrimp and brown shrimp might be up to 5 mm, with 70% of the total MPs being in the size range of 1–5 mm. The larger size of MPs accumulated in the gastrointestinal systems of sea creatures was also reported previously in [43]. The results obtained in this study indicate that MPs smaller than 250 µm were easily permeable in the gastrointestinal systems and accumulated in the tissues of shrimp, while MPs larger than 500 µm tended to accumulate in the gastrointestinal systems, due to the difficulty of their permeation through these gastrointestinal systems. 

### 3.3. Polymer Composition of MPs

Our study discovered six types of polymers, including rayon, polyethylene terephthalate, polystyrene, polyacrylic, polyamide, and polyethylene, in the shrimps collected from Cau Hai Lagoon. These polymers have been frequently detected in other research studies on shrimps. In one such study conducted by Yan et al. [32] on farmed *Litopenaeus vannamei* shrimp from Guangdong Province, China, cellulose (67.9%) was found to be the primary polymer in MPs, followed by PE. Similarly, Severini et al. [31] reported that the fibers found in wild *Phalacrognathus muelleri* shrimp comprised PE, PP, and cellulose. Another study on *Paratya australiensis* by Nan et al. [26] identified 11 types of polymers, with rayon being the most common (22.6%), followed by PES (7.5%). Hossain et al. [16] detected PA (59.1%) and rayon (27.3%) in two wild species of penaeid shrimp (*Metapenaeus monoceros* and *Penaeus monodon*) from the Bay of Bengal. In our study, rayon was found to be the most common MP (61.4%), followed by polyacrylic (11.4%), polystyrene (9.1%), and polyamide, which was found with a prevalence of 6.8%, and finally, polyethylene at 4.5%. The FTIR spectra of rayon, PET, polyamide, polyethylene, polyacrylic, and polystyrene were shown in Figure 3.

Recently, many studies have claimed that rayon fiber was found as a microplastic in fish tissue [8,37,44] or shellfish tissue [45]. On the other hand, although artificial fibers such as viscose, cellulose acetate, and rayon might contain petroleum-based materials, they are not entirely produced [46]. Nevertheless, washing textiles made with these fibers could result in the discharge of these substances into the environment, which is presently regarded as a growing threat to the marine ecosystem [47,48].

## 4. Materials and Method

### 4.1. Chemicals

Both potassium hydroxide (KOH, 45 wt.% in H_2_O) and sodium iodide (NaI, No. 383112, ACS reagent, ≥99.5%) were used for digesting the samples and extracting the micro debris. They were purchased from Sigma-Aldrich. Glass fiber filter (GF/B, No.1821-047, Whatman, Maidstone, UK) with a 47 mm diameter was used in this study during the sample processing procedure. The pure pellets of polypropylene (PP), polyethylene (PE), polyethylene terephthalate (PET), polyamide (PA), and polycarbonate (PC), which were used as microplastic standards, were purchased from Sigma Aldrich (St. Louis, MO, USA). The PP, PA, and PC were about 3–4 mm in size, the PET was about 1 mm in size, and the PE was less than 50 µm in size. 

### 4.2. Sample Collection and Processing

**Figure 4:**
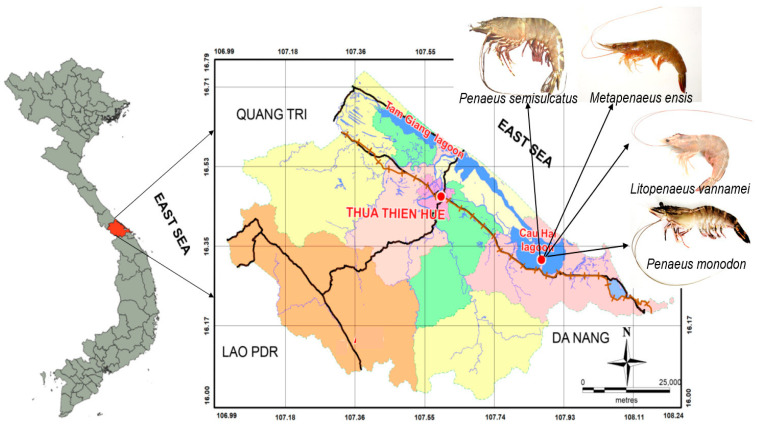
Map of the study area.

The selected samples for the analysis were several types of shrimp, according to the following priority principles: (i) are widely consumed by people, (ii) are typical for the shrimp species in the study area, and (iii) are of natural origin and aquaculture. The samples of the farmed and wild-caught shrimps were obtained in August 2022 from Cau Hai Lagoon, central Vietnam (Figure 4). The farmed shrimp samples were collected in the harvest season (commercial samples), including white-leg shrimp (*Litopenaeus vannamei*), and giant tiger shrimp (*Penaeus monodon*) from two typical shrimp farms in Cau Hai Lagoon. The wild-caught shrimp samples, including greasy-back shrimp (*Metapenaeus ensis*) and green tiger shrimp (*Penaeus semisulcatus*), were collected on fisherman’s boats after their fishing trip. The sampling location coordinates are presented in Table S1. Relevant information on the numbers and characteristics of each species are summarized in Table 3. All the samples were kept in cool containers with ice during transportation to the laboratory, where they were then analyzed as soon as possible. The gastrointestinal tract (GT) and tissue samples were performed separately for the MP analysis. The specimens were dissected separately on a metal tray using scissors, a scalpel, and forceps, and the gastrointestinal tracts were put into Petri dishes, weighed, and transferred into 125 mL glass flasks with a stopper for the next procedure.

### 4.3. Samples Preparation, Digestion, and Observation

To the best of our knowledge, four major chemical groups have been used to degrade biological matrices, including acids, bases, strong oxidizing agents, and enzymes, for extracting microplastics [9,10,49]. However, Van Cauwenberghe et al. [9] also demon-strated that polystyrene (PS) particles were deformed when contacted with HNO_3_. Simi-larly, Avio et al. [5] found degradations of PE and PS in the digestive system of *M. cephalus* when using a 22.5 M HNO_3_ digested solution. Furthermore, Karami et al. [42] reported that a digestion solution such as H_2_O_2_ (35%), NaOH (5 M), HCl (5% or 37%), HNO3 (5% or 69%), or NaClO (5%) did not give high digestion efficiencies (<95%) for extracting the MPs from fish samples. On the other hand, microscopy is used to determine physical properties such as shape, color, or size. Decomposing samples using strong acids or acids with strong oxidizing properties at high concentrations can cause discoloration or physical changes in microplastics. Due to the good performance of KOH in our previous research [7,28], KOH 10% (*w*/*v*) was chosen to digest the shrimp samples in this study. After KOH (10%) was added into a flask containing the samples, with a ratio of 10 mL of KOH per 1 g-ww of the sample [42], the flask was then wrapped in aluminum foil and kept at 40 °C for 48 h, followed by being kept for 24 h in ambient conditions. Subsequently, the flask containing the digested material was filled with 30 mL of filtered, saturated NaI solution to separate the high-density components from the whole-tissue extracts [25]. The liquid was then mixed in an ultrasonic bath (Power-Sonic 420, Korea) for 30 min and allowed to settle for 60 min. The supernatant containing MPs was collected by filtering the liquid through a microfiber filter (Whatman GF/B) using a vacuum system. The remaining parts continued to dissolve with NaI to separate the supernatant through the filter membrane. This process was repeated three times. The overlying water was directly filtered over a 1.0 μm pore size 47 mm diameter glass microfiber filter (Whatman GF/B) with a vacuum system. Finally, the filter was placed into a clean Petri dish with a cover for further measurements. 

### 4.4. Identification of Microplastics

Under a Biocular Stereo Microscope (YM0745-L) equipped with a 5.0-megapixel camera, the filter paper containing the extracted microplastics was observed. A heated needle was used to distinguish the MPs and other particles (sand or undissolved shrimp tissues), as described in a previous study [49]. When a heated needle touched the extracted items, if an item was microplastic, it would emit an odor of smelting and become curly, especially if it was a fiber. No specific phenomenon was found as the hot needle pointed at sandy items. The morphology properties of the MPs were classified into fiber, fragment, and pellet and confirmed according to their physical characteristics. The size of the MPs was measured using a calibrated stage micrometer scale and ocular scale [16]. The MPs were classified into five sizes, <100 μm, 100–250 μm, 250–500 μm, 500–1000 μm, and 1–5 mm. The MPs were categorized into five different colors, including white-transparent, yellow-orange, red-pink, blue-green, and black-grey. In total, 105 items (about 39% of the total MPs) from four shrimp species were randomly selected and verified for their chemical compositions using an iS50 FTIR system with an integrated ATR diamond crystal. 

All the detailed information regarding the methods for avoiding contamination in the laboratory, performing blanks, controlling the sample degradation efficiency, and evaluating the recovery of the analytical methods is presented in the quality assurance and quality control (QA/QC) section, which includes in the Supporting Information (SI).

## 5. Conclusions

As one of the first investigations on the MPs in shrimps collected from Cau Hai Lagoon, central Vietnam, this study successfully separated and evaluated the occurrences of MPs in the tissues and GTs of wild and farmed shrimps. The results indicated that MPs were found in the commercial shrimp collected from Cau Hai Lagoon, suggesting that the occurrence of MPs in seafood is an unneglectable and possible risk for humans consuming shrimps for daily food. The concentration of microplastics in the GT samples was significantly higher than that in the tissue samples (*p* < 0.05). MPs smaller than 250 µm were mainly found in the tissue samples, while the size of the MPs in the GT samples was significantly larger than that in the tissue samples, suggesting that the permeation of MPs through GT systems might be influenced by the size of these MPs. The number and detection frequency of the microplastics found in the farmed shrimps (white-leg shrimp and giant tiger shrimp) were statistically significantly higher than the number of microplastics in the wild-caught shrimps (greasy-back shrimp and green tiger shrimp) (*p* < 0.05), indicating that shrimp species, living environment, and microplastic contamination of feeding might affect the accumulation of these MPs in shrimps. A fibrous shape and rayon were the most common shape and polymer found in the MPs found in the tissue and GT samples, suggesting that the main sources of microplastics might be wastewater from washing clothes containing many man-made fibers. Other polymers, such as polyamide, PET, polyethylene, polystyrene, and polyacrylic, imply that various sources contributed to the occurrence of the MPs in these samples. Further studies should be conducted to determine if the features of a species or the pollution level of the surrounding environment leads to such a specificity in shrimps in particular and in seafood in general.

## Figures and Tables

**Table 1:** Comparison of microplastic pollution in shrimps in the present study with those in previous studies.

Specie	Type of Sample	Levels of MPs (Items/Individual)	Levels of MPs (Items/g-ww)	Size (μm)	Microplastic Type	Nation	Reference
Greasy-back shrimp (*Metapenaeus ensis*)	Whole	2.5 ± 0.5 ^1^	0.7 ± 0.3	<100–1000	Rayon, PET, Polyethylene, Polyamide, Polystyrene, Polyacrylic	Cau Hai Lagoon, Central Vietnam	This study
	GT	0.9 ± 0.2	2.1 ± 0.3	<100–1000			
	Tissue	1.0 ± 0.8	0.5 ± 0.4	<100–250			
Green tiger shrimp (*Penaeus semisulcatus*)	Whole	2.3 ± 0.7 ^1^	0.6 ± 0.2	<100–1000	Rayon, PET, Polyethylene, Polyamide, Polystyrene, Polyacrylic		
	GT	1.4 ± 0.3 *	3.2 ± 0.7	<100–500			
	Tissue	0.9 ± 0.8 *	0.3 ± 0.3	<100–500			
White-leg shrimp (*Litopenaeus vannamei*)	Whole	8.6 ± 3.5 ^2^	1.1 ± 0.4	<100–1000	Rayon, PET, Polyethylene, Polyamide		
	GT	6.3 ± 1.4 **	104 ± 73	<100–1000			
	Tissue	2.3 ± 0.4 **	0.3 ± 0.1	<100–500			
Giant tiger shrimp (*Penaeus monodon*)	Whole	7.7 ± 3.5 ^2^	0.5 ± 0.3	<100–2000	Rayon, PET, Polyethylene, Polyamide		
	GT	5.4 ± 1.3 ***	28.3 ± 5.7	<100–1000			
	Tissue	2.3 ± 0.7 ***	0.2 ± 0.1	<100–500			
*Crangon crangon*	Whole body	1.23 ± 0.99	0.68 ± 0.55	200–1000	Fibers	Southern North Sea	Devriese et al. [15]
*Crangon allmanni*	Whole body	1–3	-	-	Fragments and films of polyethylene and polyacrylic	Jeløya, Norway	Bour et al. [23]
*Penaeus semisulcatus*	Muscle	0.36	-	50–5000 μm fibers and <50 μm fragments	Fibers and fragments	Persian Gulf	Akhbarizadeh et al. [24]
*Fenneropenaeus indicus*	Whole body	0.04 ± 0.07	-	157–2785	Fibers, fragments and films of polyethylene, polypropylene and polyamide	Kochi, India	Daniel et al. [25]
*Paratya australiensis*	-	24 ± 31	-	<1–2 mm	Fibers of rayon and polyester	Northern central Victoria, Australia	Nan et al. [26]
*Penaeus monodon*	GT	6.6 ± 2	3.40 ± 1.23	250–500	Fibers, particles and fragments of polyamide 6 and rayon	Northern Bay of Bengal, Bangladesh	Hossain et al. [16]
*Metapenaeus monoceros*		7.8 ± 2	3.87 ± 1.05	1000–5000			
*Litopenaeus vannamei*, *Pleoticus muelleri*, *Fenneropenaeus indicus*	GT	21.0 ± 4.0	-	-	Films, fibers, fragments and spheres	Singapore	Curren et al. [17]
*Metapenaeus monoceros*, *Parapeneopsis stylifera*, *Penaeus indicus*	GT	6.78 ± 2.80	70.32 ± 34.67	100–250	Fibers, fragments, beads, pellets and films of polyethylene, polypropylene, polyethylene terephthalate, polyester and polyamide	Arabian Sea	Gurjar et al. [18]
*Litopenaeus vannamei*	Whole	18.5 ± 1.2	1.06	<2000		Northwestern Mexico	Valencia-Castañeda et al. [27]
	GT	7.6 ± 0.6	261.7 ± 84.5				
	Gills	6.3 ± 0.9	13.1 ± 1.8				
	Exoskeleton	4.3 ± 0.9	2.6 ± 0.6				

Note: ^1^ and ^2^ indicate significant difference of MPs observed in the whole wild and farmed shrimps (the different number for *p* < 0.05); *, **, and *** represent the significant difference (*p* < 0.05) in MPs measured in GT and tissue samples of green tiger shrimp, white-leg shrimp, and giant tiger shrimp, respectively.

**Table 2:** Chemical composition of microplastics found in four types of shrimp.

Description	Number of MPs					Percentage (%)
	Greasy-Back *Metapenaeus ensis*	Green Tiger *Penaeus semisulcatus*	White-Leg *Litopenaeus vannamei*	Giant Tiger *Penaeus monodon*	Total Items	
Total selected items	31	25	24	23	105	100%
Rayon	18	18	14	15	65	61.9%
PET	2	-	3	2	7	6.7%
Polyethylene	1	1	2	2	6	5.7%
Polyamide	2	2	3	4	11	10.5%
Polystyrene	3	1	-	-	4	3.8%
Polyacrylic	3	2	-	-	5	4.8%
Unidentified	2	1	2	2	7	6.7%

**Table 3:** Lengths and weighst of shrimp from Cau Hai Lagoon, central Vietnam.

Common Name	Species	Habitat	Number of Sample	Shell Length (cm)	Shell Width (cm)	Shell Weight (g/Individual)	Soft Tissue Weight (g/Individual)
Greasy-back shrimp	*Metapenaeus ensis*	Wild	30	8.2 ± 0.3	2.0 ± 0.1	3.6 ± 0.6	2.6 ± 0.4
Green tiger shrimp	*Penaeus semisulcatus*	Wild	30	10.4 ± 0.7	1.4 ± 0.3	8.2 ± 1.2	4.4 ± 1.0
White-leg shrimp	*Litopenaeus vannamei*	Farmed	30	13.8 ± 0.8	3.0 ± 0.1	14.4 ± 2.8	8.1 ± 0.8
Giant tiger shrimp	*Penaeus monodon*	Farmed	30	17.1 ± 1.0	3.0 ± 0.1	32.4 ± 3.1	14.9 ± 0.8

## Data Availability

Not applicable.

## Supplementary Materials

The following supporting information can be downloaded at: https://www.mdpi.com/article/10.3390/molecules28124634/s1, Table S1: Sampling location coordinates in this study. Refs. [50,51] are cited in Supplementary Materials.

## References

[1] Eriksen M., Lebreton L.C., Carson H.S., Thiel M., Moore C.J., Borerro J.C., Reisser J. (2014). Plastic pollution in the world’s oceans: More than 5 trillion plastic pieces weighing over 250,000 tons afloat at sea. PLoS ONE.

[2] PlasticEurope (2016). Plastics—The Facts 2016. https://www.plasticseurope.org/application/files/4315/1310/4805/plastic-the-fact-2016.pdf.

[3] Lebreton L.C., Van der Zwet J., Damsteeg J.W., Slat B., Andrady A., Reisser J. (2017). River plastic emissions to the world’s oceans. Nat. Commun..

[4] Barboza L.G.A., Vethaak A.D., Lavorante B.R., Lundebye A.-K., Guilhermino L. (2018). Marine microplastic debris: An emerging issue for food security, food safety and human health. Mar. Pollut. Bull..

[5] Avio C.G., Gorbi S., Milan M., Benedetti M., Fattorini D., d’Errico G., Pauletto M., Bargelloni L., Regoli F. (2015). Pollutants bioavailability and toxicological risk from MP to marine mussels. Environ. Pollut..

[6] Avio C.G., Gorbi S., Regoli F. (2017). Plastics and MP in the oceans: From emerging pollutants to emerged threat. Mar. Environ. Res..

[7] My T.T.A., Dat N.D., Hung N.Q., Quang D.T. (2023). Preliminary determination of microplastics in bivalves collected from Phu Yen, central Viet Nam. Vietnam. J. Sci. Technol..

[8] Bessa F., Barría P., Neto J.M., Frias J.P.G.L., Otero V., Sobral P., Marques J.C. (2018). Occurrence of microplastics in commercial fish from a natural estuarine environment. Mar. Pollut. Bull..

[9] Van Cauwenberghe L., Claessens M., Vandegehuchte M.B., Janssen C.R. (2015). Microplastics are taken up by mussels (*Mytilus edulis*) and lugworms (*Arenicola marina*) living in natural habitats. Environ. Pollut..

[10] Van Cauwenberghe L., Janssen C. (2014). Microplastics in bivalves cultured for human consumption. Environ. Pollut..

[11] Brennecke D., Duarte B., Paiva F., Caçador I., Canning-Clode J. (2016). Microplastics as vector for heavy metal contamination from the marine environment. Estuar. Coast. Shelf Sci..

[12] Bradney L., Wijesekara H., Palansooriya K.N., Obadamudalige N., Bolan N.S., Ok Y.S., Kirkham M.B. (2019). Particulate plastics as a vector for toxic trace-element uptake by aquatic and terrestrial organisms and human health risk. Environ. Int..

[13] Jambeck J.R., Geyer R., Wilcox C., Siegler T.R., Perryman M., Andrady A., Narayan R., Law K.L. (2015). Plastic waste inputs from land into the ocean. Science.

[14] Lahens L., Strady E., Kieu-Le T.-C., Dris R., Boukerma K., Rinnert E., Gasperi J., Tassin B. (2018). Macroplastic and microplastic contamination assessment of a tropical river (Saigon River, Vietnam) transversed by a developing megacity. Environ. Pollut..

[15] Devriese L.I., van der Meulen M.D., Maes T., Bekaert K., Paul-Pont I., Frère L., Robbens J., Vethaak A.D. (2015). Microplastic contamination in brown shrimp (*Crangon crangon*, Linnaeus 1758) from coastal waters of the Southern North Sea and Channel area. Mar. Pollut. Bull..

[16] Hossain M.S., Rahman M.S., Uddin M.N., Sharifuzzaman S., Chowdhury S.R., Sarker S., Chowdhury M.S.N. (2020). Microplastic contamination in Penaeid shrimp from the Northern Bay of Bengal. Chemosphere.

[17] Curren E., Leaw C.P., Lim P.T., Leong S.C.Y. (2020). Evidence of Marine Microplastics in Commercially Harvested Seafood. Front. Bioeng. Biotechnol..

[18] Gurjar U.R., Xavier M., Nayak B.B., Ramteke K., Deshmukhe G., Jaiswar A.K., Shukla S.P. (2021). Microplastics in shrimps: A study from the trawling grounds of north eastern part of Arabian Sea. Environ. Sci. Pollut. Res..

[19] Teuten E.L., Saquing J.M., Knappe D.R., Barlaz M.A., Jonsson S., Björn A., Rowland S.J., Thompson R.C., Galloway T.S., Yamashita R. (2009). Transport and release of chemicals from plastics to the environment and to wildlife. Philos. Trans. R. Soc. B Biol. Sci..

[20] Suman T.Y., Jia P.P., Li W.G., Junaid M., Xin G.Y., Wang Y., Pei D.S. (2020). Acute and chronic effects of polystyrene microplastics on brine shrimp: First evidence highlighting the molecular mechanism through transcriptome analysis. J. Hazard. Mater..

[21] Bouwmeester H., Hollman P.C.H., Peters R.J.B. (2015). Potential health impact of environmentally released micro- and nano-plastics in the human food production chain: Experiences from nanotoxicology. Environ. Sci. Technol..

[22] Giuliani S., Romano S., Turetta C., Cu N.H., Bellucci L.G., Capodaglio G., Mugnai C., Nhon D.H., Frignani M. (2011). Soils and sediments of the Thua Thien-Hue Province (central Vietnam): Recognizing trace element sources and the likely influence of natural events. J. Environ. Monit..

[23] Bour A., Avio C.G., Gorbi S., Regoli F., Hylland K. (2018). Presence of microplastics in benthic and epibenthic organisms: Influence of habitat, feeding mode and trophic level. Environ. Pollut..

[24] Akhbarizadeh R., Moore F., Keshavarzi B. (2019). Investigating microplastics bioaccumulation and biomagnification in seafood from the Persian Gulf: A threat to human health?. Food Addit. Contam. Part A.

[25] Daniel D.B., Ashraf P.M., Thomas S.N., Thomson K.T. (2021). Microplastics in the edible tissues of shellfishes sold for human consumption. Chemosphere.

[26] Nan B., Su L., Kellar C., Craig N.J., Keough M.J., Pettigrove V. (2020). Identification of microplastics in surface water and Australian freshwater shrimp *Paratya australiensis* in Victoria, Australia. Environ. Pollut..

[27] Valencia-Castañeda G., Ruiz-Fernández A.C., Frías-Espericueta M.G., Rivera-Hernández J.R., Green-Ruiz C.R., Páez-Osuna F. (2022). Microplastics in the tissues of commercial semi-intensive shrimp pond-farmed *Litopenaeus vannamei* from the Gulf of California ecoregion. Chemosphere.

[28] My T.T.A., Dat N.D., Long H.T., Binh T.T. (2022). Occurrence of microdebris in muscle of round scad (*Decapterus maruadsi*) collected from Central Vietnam. Environ. Asia.

[29] Fang C., Zheng R., Hong F., Jiang Y., Chen J., Lin H. (2021). Microplastics in three typical benthic species from the Arctic: Occurrence, characteristics, sources, and environmental implications. Environ. Res..

[30] Wang T., Tong C., Wu F., Jiang S., Zhang S. (2023). Distribution characteristics of microplastics and corresponding feeding habits of the dominant shrimps in the rivers of Chongming Island. Sci. Total Environ..

[31] Severini M.F., Buzzi N.S., López A.F., Colombo C.V., Sartor G.C., Rimondino G.N., Truchet D.M. (2020). Chemical composition and abundance of microplastics in the muscle of commercial shrimp *Pleoticus muelleri* at an impacted coastal environment (Southwestern Atlantic). Mar. Pollut. Bull..

[32] Yan M., Li W., Chen X., He Y., Zhang X., Gong H. (2021). A preliminary study of the association between colonization of microorganism on microplastics and intestinal microbiota in shrimp under natural conditions. J. Hazard. Mater..

[33] Wehrheim C., Lübken M., Stolpe H., Wichern M. (2023). Identifying Key Influences on Surface Water Quality in Freshwater Areas of the Vietnamese Mekong Delta from 2018 to 2020. Water.

[34] Murphy C.L. (2018). A Comparison of Microplastics in Farmed and Wild Shellfish near Vancouver Island and Potential Implications for Contaminant Transfer to Humans. Ph.D. Thesis.

[35] Yin J., Li J.-Y., Craig N.J., Su L. (2022). Microplastic pollution in wild populations of decapod crustaceans: A review. Chemosphere.

[36] Atamanalp M., Köktürk M., Uçar A., Duyar H.A., Özdemir S., Parlak V., Esenbuğa N., Alak G. (2021). Microplastics in Tissues (Brain, Gill, Muscle and Gastrointestinal) of *Mullus barbatus* and *Alosa immaculata*. Arch. Environ. Contam. Toxicol..

[37] Güven O., Gökdağ K., Jovanović B., Kıdeyş A.E. (2017). Microplastic litter composition of the Turkish territorial waters of the Mediterranean Sea, and its occurrence in the gastrointestinal tract of fish. Environ. Pollut..

[38] Lusher A.L., Burke A., O’connor I., Officer R. (2014). Microplastic pollution in the Northeast Atlantic Ocean: Validated and opportunistic sampling. Mar. Pollut. Bull..

[39] Neves D., Sobral P., Ferreira J.L., Pereira T. (2015). Ingestion of microplastics by commercial fish off the Portuguese coast. Mar. Pollut. Bull..

[40] Bellas J., Martínez-Armental J., Martínez-Cámara A., Besada V., Martínez-Gómez C. (2016). Ingestion of microplastics by demersal fish from the Spanish Atlantic and Mediterranean coasts. Mar. Pollut. Bull..

[41] Compa M., Ventero A., Iglesias M., Deudero S. (2018). Ingestion of microplastics and natural fibers in *Sardina pilchardus* (Walbaum, 1792) and *Engraulis encrasicolus* (Linnaeus, 1758) along the Spanish Mediterranean coast. Mar. Pollut. Bull..

[42] Karami A., Golieskardi A., Choo C.K., Romano N., Ho Y.B., Salamatinia B. (2017). A high-performance protocol for extraction of MP in fish. Sci. Total Environ..

[43] Abbasi S., Soltani N., Keshavarzi B., Moore F., Turner A., Hassanaghaei M. (2018). Microplastics in different tissues of fish and prawn from the Musa Estuary, Persian Gulf. Chemosphere.

[44] Savoca S., Capillo G., Mancuso M., Faggio C., Panarello G., Crupi R., Bonsignore M., D’Urso L., Compagnini G., Neri F. (2019). Detection of artificial cellulose microfibers in Boops boops from the northern coasts of Sicily (Central Mediterranean). Sci. Total Environ..

[45] Chinfak N., Sompongchaiyakul P., Charoenpong C., Shi H., Yeemin T., Zhang J. (2021). Abundance, composition, and fate of microplastics in water, sediment, and shellfish in the Tapi-Phumduang River system and Bandon Bay, Thailand. Sci. Total Environ..

[46] Cesa F.S., Turra A., Baruque-Ramos J. (2017). Synthetic fibers as microplastics in the marine environment: A review from textile perspective with a focus on domestic washings. Sci. Total Environ..

[47] Macieira R.M., Oliveira L.A.S., Cardozo-Ferreira G.C., Pimentel C.R., Andrades R., Gasparini J.L., Sarti F., Chelazzi D., Cincinelli A., Gomes L.C. (2021). Microplastic and artificial cellulose microfibers ingestion by reef fishes in the Guarapari Islands, southwestern Atlantic. Mar. Pollut. Bull..

[48] Suaria G., Achtypi A., Perold V., Lee J.R., Pierucci A., Bornman T.G., Aliani S., Ryan P.G. (2020). Microfibers in oceanic surface waters: A global characterization. Sci. Adv..

[49] Nuelle M.-T., Dekiff J.H., Remy D., Fries E. (2014). A new analytical approach for monitoring microplastics in marine sediments. Environ. Pollut..

[50] Digka N., Tsangaris C., Torre M., Anastasopoulou A., Zeri C. (2018). Microplastics in mussels and fish from the Northern Ionian Sea. Mar. Pollut. Bull..

[51] Cole M., Webb H., Lindeque P.K., Fileman E.S., Halsband C., Galloway T.S. (2014). Isolation of microplastics in biota-rich seawater samples and marine organisms. Sci. Rep..

