# Supplementary material for: Occurrence and Characteristics of Microplastics in Wild and Farmed Shrimps Collected from Cau Hai Lagoon, Central Vietnam
Source: Molecules. 2023 Jun 8;28(12):4634. doi: 10.3390/molecules28124634 (PMC10305481; doi:10.3390/molecules28124634)
Supplement: Supplementary file 1 [file molecules-28-04634-s001.zip › molecules-2446295-supplementary.pdf]

SUPPORTING INFORMATION

Occurrence and Characteristics of Microplastics in Wild- and Farmed- Shrimps Collected from Cau Hai Lagoon, Central Vietnam

Tran Thi Ai My[1,*], Nguyen Duy Dat[2], Nguyen Quoc Hung[3]

[1] *Department of Chemistry, University of Sciences, Hue University, Hue 53000, Vietnam*

[2]*Faculty of Chemical & Food Technology, Ho Chi Minh City University of Technology and Education, Thu Duc, Ho Chi Minh 70000, Vietnam*

[3]*Center of Analytical Sevices and Experimentation HCMc, Ho Chi Minh 70000, Vietnam*

Quality assurance and quality control (QA/QC)

To avoid microplastic contamination during sample processing and analysis, a solution of 70% ethanol was frequently used to clean the work area [1, 2]. All equipment and apparatuses used for the experiments were made of glass and metal. Laboratory clothes and gloves used during the experiments were cotton and nitrile gloves, respectively. A procedural blank extraction without tissue was carried out in parallel with the fish tissue samples to control contamination during the experimental procedure (5 blank samples were used for one batch of samples). Processing contamination must be less than 5% of the average number of microplastics in the sample [3]. During the handling of the samples, the contamination with airborne microplastics was prevented and the procedural blanks only contained 0.2 ± 0.4 item/filter. In order to avoid contamination of the samples from airborne microplastics, the NaI and KOH solutions were filtered with Whatman filter paper GF/B prior to use. The containers and beakers were rinsed with filtered DI water three times and covered until use. The samples were carefully wrapped with aluminum foil if not in use. All the experimental procedures were finished as soon as possible.

The efficiency of fish tissue digestion was tested by calculating the amount of undigested organic and/or inorganic materials remaining on the filter. The digestion efficiency H (%) was calculated according to Eq. (1). The efficiency of this digestion method is very high, reaching 96% to 102%, within the range of optimum digestion efficiency reported in previous studies [4, 5].

$$H\,(\%) = \frac{m_S - (m_a - m_0)}{m_S} \times 100 \qquad (1)$$

where m_S is the initial weight of the tissue sample; m_a is the weight of dry filter paper after filtration; m_0 is the weight of dry filter paper before filtration.

The accuracy of the analytical procedure was tested via the analysis of standardized polymers. For controlling the influence of the digestion method on the polymers (degradation, deformation, color change) and calculating the efficiency of polymer flotation of the NaI solution after sample digestion, the standards of polymers (PP, PE, PET, PA, PC) were added to the fish tissue samples before digestion. The recovery was calculated according to Eq. (2), and the value for PP, PE, PET, PA, PC ranges from 95% to 104%.

$$Rev\,(\%) = \frac{m_a - m_0}{m_{Spiked\ MPs}} \times 100 \qquad (2)$$

where m_a and m_0 are described previously, and $m_{\text{spiked MPs}}$ is the mass of the polymer added as standards (PP, PE, PET, PA, PC).

REFERENCES:

1. Karami, A., Golieskardi, A., Choo, C. K., Romano, N., Ho, Y. B., & Salamatinia, B., 2017. A high-performance protocol for extraction of MP in fish". *Sci. Total Environ.* 578, 485-494.
2. My, T. T. A., Dat, N. D., Long, H. T., & Binh, T. T., 2022. Occurrence of microdebris in muscle of round scad (Decapterus ma-ruadsi) collected from Central Vietnam. EnvironmentAsia. 15(3) (2022), 38-47.

3. Digka, N., Tsangaris, C., Torre, M., Anastasopoulou, A., & Zeri, C., 2018. Microplastics in mussels and fish from the Northern Ionian Sea. Mar. Pollut. Bull. 135, 30-40.

4. Cole, M., Webb, H., Lindeque, P. K., Fileman, E. S., Halsband, C., & Galloway, T. S., 2014. Isolation of microplastics in biota-rich seawater samples and marine organisms. Sci. Rep. 4(1), 1–8.

5. Karami, A., Golieskardi, A., Choo, C. K., Romano, N., Ho, Y. B., & Salamatinia, B., 2017. A high-performance protocol for extraction of microplastics in fish. Sci. Total Environ. 578, 485–494.

Table S1. Sampling location coordinates in this study

Species	X	Y
Greasy-back shrimp	107.8659973	16.31870079
Green tiger shrimp	107.8789978	16.31800079
White-leg shrimp	107.8820038	16.34600067
Giant tiger shrimp	107.8700027	16.34720039
